# Characteristic Curves of the Lennard-Jones Fluid

**DOI:** 10.1007/s10765-020-02721-9

**Published:** 2020-08-20

**Authors:** Simon Stephan, Ulrich K. Deiters

**Affiliations:** 1 Laboratory of Engineering Thermodynamics (LTD), TU Kaiserslautern, Erwin-Schrödinger-Straße 44, 67663 Kaiserslautern, Germany; 2 Institute of Physical Chemistry, University of Cologne, Greinstraße 4-6, 50939 Cologne, Germany

**Keywords:** Lennard-Jones fluid, Equation of state, Characteristic curves, Virial coefficients

## Abstract

Equations of state based on intermolecular potentials are often developed about the Lennard-Jones (LJ) potential. Many of such EOS have been proposed in the past. In this work, 20 LJ EOS were examined regarding their performance on Brown’s characteristic curves and characteristic state points. Brown’s characteristic curves are directly related to the virial coefficients at specific state points, which can be computed exactly from the intermolecular potential. Therefore, also the second and third virial coefficient of the LJ fluid were investigated. This approach allows a comparison of available LJ EOS at extreme conditions. Physically based, empirical, and semi-theoretical LJ EOS were examined. Most investigated LJ EOS exhibit some unphysical artifacts.

**Electronic supplementary material:**

The online version of this article (10.1007/s10765-020-02721-9) contains supplementary material, which is available to authorized users.

## Introduction

The Lennard-Jones (12,6) potential [1, 2] has been extensively used since the early days of computer simulation [3–6] for the modeling of repulsive and dispersive interactions of simple fluids. It is probably the most frequently investigated monomer model fluid in molecular simulation [7]. The Lennard-Jones (LJ) potential can be favorably used for testing new theories and simulation methods, e.g., for mixtures, phase changes, non-equilibrium phenomena, and interfaces between phases [8–19]. Also, the Lennard-Jones potential is often used as a starting point for the development of many state-of-the-art force fields for complex molecules [20–22].

The Lennard-Jones potential is defined as

1 $$\begin{aligned} u_\text {LJ}(r) = 4 \varepsilon \left[ \left( \dfrac{\sigma }{r}\right) ^{12}-\left( \dfrac{\sigma }{r}\right) ^6\right] \, , \end{aligned}$$

where $\varepsilon$ and $\sigma$ are the energy and size parameter, respectively. The distance between two particles is denoted by *r*. Different versions of the LJ potential are used in the literature depending on the treatment of the long–range interactions, which has an important influence on the thermodynamic properties [23–32]. The present work is limited to the ’full’ Lennard-Jones potential, i.e., including long-range correction schemes [33].

Analytical model functions of the LJ fluid for the description of the thermodynamic properties, i.e., equations of state (EOS), are crucial for many applications, e.g., the development of theories for more complex fluids like polymers, electrolyte solutions, and associating fluids. LJ EOS have been used successfully as base models for a *reference fluid* to describe more complex fluids [34–38]. A large number of equations of state have been proposed for the LJ fluid, of which 20 are studied here, cf. Table 1.

**Table 1:** Lennard-Jones equations of state used in the present work—sorted chronologically

Authors	Abbr.	EOS type	Year
Nicolas et al. [51]	Ni	Empirical (MBWR); pressure explicit	1979
Ree [53]	Re	Empirical; pressure explicit	1980
Cotterman et al. [35]	Co	BH perturbation theory + virial; Helmholtz energy explicit	1986
Adachi et al. [64]	Ad	Empirical (MBWR); pressure explicit	1988
Koutras et al. [66]	Kou	Modified HS equation; pressure explicit	1992
Miyano [61]	Mi	Empirical (MBWR); pressure explicit	1993
Johnson et al. [28]	Jo	Empirical (MBWR); pressure explicit	1993
Kolafa and Nezbeda [52]	Ko	HS + virial + empirical; Helmholtz energy explicit	1994
Mecke et al. [54, 55]	Me	HS + empirical; Helmholtz energy explicit	1996
Sun and Teja [60]	Su	Empirical (MBWR); pressure explicit	1996
Hess [67]	He	WCA reference + virial; pressure explicit	1999
Boltachev and Baidakov [68]	Bo	Empirical + virial; pressure explicit	2003
Paricaud [57]	Pa	BH perturbation theory; Helmholtz energy explicit	2006
Quiñones-Cisneros et al. [65]	Qui	Empirical; pressure explicit	2009
May and Mausbach [62, 63]	Ma	Empirical (MBWR); pressure explicit	2012
Lafitte et al. [38]	La	BH perturbation theory; Helmholtz energy explicit	2013
Thol et al. [41]	Th	Empirical; Helmholtz energy explicit	2016
van Westen and Gross [56]	vWe	BH perturbation theory; Helmholtz energy explicit	2017
Gottschalk [69]	Go	Empirical; virial coefficients; Helmholtz energy explicit	2019
Stephan et al. [46]	St	BH perturbation theory; Helmholtz energy explicit	2020

The columns are: authors, abbreviation for EOS, EOS type, and year of publication

Lennard-Jones EOS can be broadly separated into two types: empirical EOS and physically motivated EOS. ‘Physically motivated’ EOS means that the employed functions were derived from statistical mechanics, whereas an empirical EOS is a correlation of computer experiment data by a convenient but arbitrary mathematical form. However, the distinction between both types is not sharp; most LJ EOS have some physically motivated features and some empirical features, cf. Deiters and de Reuck [39] for a detailed discussion. It is widely accepted that empirical EOS have a tendency to exhibit poor extrapolation behavior to fluid regions and physical properties that were not considered during the parametrization and may even yield a physically unreasonable behavior in some regions, e.g., multiple van der Waals loops in the vapor–liquid two phase region [40–42] or the crossing of isotherms [43–45]. Physically motivated LJ EOS on the other side are often less precise in the description of homogeneous state properties—particularly higher-order thermodynamic derivatives.

The LJ EOS of Johnson et al. [28], Lafitte et al. [38], and Stephan et al. [46] are of particular interest since those are the base in SAFT [34, 47, 48] type EOS for the modeling of repulsive and dispersive interactions, i.e., soft-SAFT [36, 37], SAFT-VR Mie [38, 49], and PC-SAFT [50], respectively. The LJ EOS of Ref. [46] is a re-parametrization of the PC-SAFT monomer model—developed to give a good description of the LJ fluid.

The most popular LJ EOS are those of Johnson et al. [28], Nicolas et al. [51], Kolafa and Nezbeda [52], Cotterman et al. [35], Lafitte et al. [38], Ree [53], and Mecke et al. [54, 55] (sorted by their the number of citations in the *Web of Science*).

Table 1 also indicates the types of the LJ EOS, i.e., whether they are Helmholtz energy or pressure explicit. We compare the performance of 20 LJ EOS, varying from purely empirical to rigorously theory-based. Physically motivated LJ EOS considered here are those of Refs. [35, 38, 46, 56, 57] and are all based on the perturbation theory of Barker and Henderson [58, 59]. LJ EOS that are empirically based are those of Refs. [28, 41, 51, 53, 60–65]. The remaining LJ EOS considered in the present work [52, 54, 55, 66–69] are denoted here as semi-theoretical.

In a recent work of our group [46], LJ EOS were systematically reviewed and compared with available computer experiment data for the LJ fluid for homogeneous state points and the vapor–liquid equilibrium. The present work pursues this comparison for a detailed discussion of Brown’s characteristic curves [70] and important characteristic states. As Brown’s characteristic curves are directly related to virial coefficients, also the second and third virial coefficient are studied. This comparison is of particular interest, since the virial coefficients of the LJ fluid can be computed exactly from their definitions in statistical mechanics, while reference data obtained from computer simulations are subject to errors and uncertainties [7, 71, 72]. Brown’s characteristic curves and the virial coefficients are directly linked in the limit of the ideal gas and therefore corporately investigated in the present work. Brown proposed the *characteristic curves* for the assessment of equations of state for a fluid with repulsive and dispersive interactions [70]. The LJ fluid is evidently an excellent candidate for such an assessment. Furthermore, Brown’s characteristic curves are an important tool for the development of new equations of state [73–75].

From Brown’s characteristic curves, the Amagat curve exhibits the largest pressure and temperature. For most gases, the Amagat curve is therefore not relevant for technical applications. Nevertheless, for particularly light-boiling gases, like neon, helium, and hydrogen, the pressure and temperature range of the Amagat curve is often relevant for technical applications. Furthermore, thermodynamic conditions in the range of the Amagat curve are relevant for fluids in geological applications as well as lubrication gaps in tribological applications. Only EOS that produce reasonable Amagat curves are appropriate for such applications.

We use reduced properties with respect to the Lennard-Jones potential throughout this article denoted by an asterisk; the definitions can be found in Table 2.

**Table 2:** Reduced properties with respect to the Lennard-Jones potential parameters $\varepsilon$ and $\sigma$ applied in the present work

Property	Symbol
Temperature	$T^* = T\,/\,(\varepsilon /k_\text {B})$
Pressure	$p^* = p\,/\,(\varepsilon /\sigma ^3)$
Density	$\rho ^* = \rho \,/\,(1/\sigma ^3)$
Internal energy	$u^* = u\,/\,\varepsilon$
Helmholtz energy	$a^* = a \,/\,\varepsilon$
Enthalpy	$h^* = h \,/\,\varepsilon$
Distance	$r^* = r \,/\,\sigma$
Volume	$V^* = V \,/\,\sigma ^3$
Specific volume	$v^* = v \,/\,\sigma ^3$
$2^{\text {nd}}$ virial coeff.	$B^* = B \,/\,(\frac{2}{3}\pi \sigma ^3)$
$3^{\text {rd}}$ virial coeff.	$C^* = C \,/\,(\frac{2}{3}\pi \sigma ^3)^2$

The properties $u^*$, $a^*$, $h^*$, $v^*$ indicate the respective quantity per particle

## Theory

Brown’s characteristic curves are defined as curves on which the compressibility factor $Z = \frac{p^{*}v^{*}}{T^{*}}$ or its derivatives match the values of an ideal gas at the same temperature and density [70]. Since EOS are usually fitted to reference data at moderate conditions, the application of the characteristic curves is often referred to as ’testing the extrapolation behavior’ of EOS [41, 73–75]. The testing of these characteristic curves has also been included in the IUPAC guidelines for publishing equations of state [76]. In this section, the definitions of Brown’s characteristic curves along with the description of the their general features are briefly outlined. Also their relation to the second and third virial coefficient $B^{*}$ and $C^{*}$, respectively, is discussed.

Brown’s characteristic curves are defined as the loci of state points at which a certain thermodynamic property of the fluid matches that of an ideal gas [70, 73, 75, 77]. Brown defined four main characteristic curves: one 0$^\text {th}$-order (named Zeno curve) and three 1$^\text {st}$-order curves (named Amagat, Boyle, and Charles curve) [70] based on the compressibility factor itself and its derivatives with respect to the temperature and pressure. For a real fluid, *Z* and its derivatives can match the values of the ideal gas for special $T^{*},v^{*}$ combinations only [70, 75], as a result of Gibbs’ phase rule. These state points collectively constitute a characteristic curve.

The characteristic curves are also known under other names: the Zeno curve as ’ideal curve’, the Amagat curve as ’Joule inversion curve’, and the Charles curve as ’Joule–Thomson inversion curve’. Here, we adopt the original naming introduced by Brown [70]. The characteristic curves are usually plotted in a double-logarithmic $p^{*}$–$T^{*}$ diagram; this convention is also adopted here. In such a diagram, the characteristic curves exhibit a typical concave dome shape, i.e., they have a negative curvature.

The Zeno, Amagat, Boyle, and Charles curve are required to have a negative curvature throughout and a single maximum in a double-logarithmic pressure–temperature diagram [70, 75]. Furthermore, Brown postulated that all four characteristic curves end in the limit of $p^{*} \rightarrow 0$ with an infinite slope in a double-logarithmic $p^{*}-T^{*}$ diagram [70]. Brown furthermore deduced that the four characteristic curves of 0$^\text {th}$ and 1$^\text {st}$-order may only contact each other at three distinct points [70, 75]: (1) the Zeno curve converges against the Amagat curve on the hypothetical extension of the vapor pressure curve; (2) the Zeno curve converges against the Boyle curve in their common limit of $p^{*} \rightarrow 0$ at $T^{*}=T_\text {Boyle}^{*}$ (the zero crossing temperature of the second virial coefficient), and (3) the Zeno curve intersects the Charles curve at the point of maximum pressure of the Zeno curve. Usually, the Amagat and Zeno curves are truncated at low temperatures by the solid–fluid equilibrium. Finally, the Amagat, Boyle, and Charles curve must not cross, but enclose each other in a $p^{*}-T^{*}$ diagram [70]: the Amagat curve surrounding the Charles curve surrounding the Boyle curve.

The characteristic curves can be computed from the Helmholtz energy per particle $a^{*}$ and its derivatives. The following notation is used for the derivatives of the Helmholtz energy with respect to the inverse temperature and density

2 $$\begin{aligned} {\tilde{a}}_{nm}^{*} = {\tilde{a}}_{nm}^\text {*id} + {\tilde{a}}_{nm}^\text {*conf} = (1/T^*)^n\, \rho ^{*m} \, \frac{\partial ^{n+m} ({\tilde{a}}^\text {*id} + {\tilde{a}}^\text {*conf})}{\partial (1/T^*)^n\, \partial \rho ^{*m}} , \end{aligned}$$

with $n,m=0,1,2$ and the tilde indicating ${\tilde{a}}^{*} = a^{*}/T^{*}$. In Eq. (2), ’id’ indicates the ideal gas contribution and ’conf’ the configurational contribution.

The density-based virial equation can be written as

3 $$\begin{aligned} Z = 1 + B^{*} \rho ^{*} + C^{*} \rho ^{*2} + \cdots . \end{aligned}$$

The Zeno curve (${\mathcal {Z}}$) is defined as the locus of state points that satisfy $Z=\frac{p^{*}v^{*}}{T^{*}} \equiv 1$ and can be computed from the Helmholtz energy as

4 $$\begin{aligned} {\tilde{a}}^{{*}\text {conf}}_{01} = 0 \,. \end{aligned}$$

Furthermore, state points on the Zeno curve have $u^{{*}\text {conf}}=0$. The Zeno curve ends at the Boyle temperature $T_{\text {Boyle}}^{*}$ in the zero-pressure limit $p^{*} \rightarrow 0$, where the third and higher virial coefficients can be neglected. This corresponds to the condition for the second virial coefficient $B^{*}(T_\text {Boyle}^{*}) = 0$, cf. Eq. (3).

The Amagat curve (${\mathcal {A}}$) is defined as the locus of state points that satisfy $\big ( \frac{\partial Z}{\partial T^{*}} \big )_{v^{*}} \equiv 0$ and can be computed from the Helmholtz energy as

5 $$\begin{aligned} {\tilde{a}}^{{*}\text {conf}}_{11} = 0 \,. \end{aligned}$$

The Amagat curve originates on the vapor pressure curve at low temperatures (if crystallization is disregarded). It ends at the Amagat temperature $T_\text {Amagat}^{*}$ in the zero-pressure limit $p^{*} \rightarrow 0$, which corresponds to the maximum of the second virial coefficient with $\text {d} B^{*} / \text {d}T^{*} = 0$. This relation can be obtained by applying $\big ( \frac{\partial Z}{\partial T^{*}} \big )_{v^{*}} = 0$ to the density-based virial equation (3) in the low-pressure limit where the third and higher virial coefficients can be neglected.

Equations of state that do not exhibit a maximum in the second virial coefficient $B^{*}(T^{*})$ have a distorted Amagat curve in the limit $p^{*} \rightarrow 0$ [70, 75].

The Boyle curve (${\mathcal {B}}$) is defined as the locus of state points that satisfy $\big ( \frac{\partial Z}{\partial 1/\rho ^{*}} \big )_{T^{*}} \equiv 0$ and can be computed from the Helmholtz energy as

6 $$\begin{aligned} {\tilde{a}}^{{*}\text {conf}}_{01} + \rho ^*{\tilde{a}}^{{*}\text {conf}}_{02} = 0 \,. \end{aligned}$$

The Boyle curve originates on the vapor pressure curve close to the critical point, runs through a pressure maximum and ends at the Boyle temperature in the limit $p^{*} \rightarrow 0$. This can be shown by applying $\big ( \frac{\partial Z}{\partial 1/\rho ^{*}} \big )_{T^{*}} = 0$ to Eq. (3) in the low-pressure limit. Hence, the Boyle and the Zeno curve converge into each other in the zero-pressure limit.

The Charles curve (${\mathcal {C}}$) is defined as the locus of state points that satisfy $\big ( \frac{\partial Z}{\partial T^{*}} \big )_{p^{*}} \equiv 0$ and can be computed from the Helmholtz energy as

7 $$\begin{aligned} {\tilde{a}}^{{*}\text {conf}}_{01} + \rho ^{*} {\tilde{a}}^{{*}\text {conf}}_{02} + 1/T^{*} {\tilde{a}}^{{*}\text {conf}}_{11} = 0 \,. \end{aligned}$$

The Charles curve—also known as Joule–Thomson inversion curve—is of fundamental technical importance as it determines the transition locus from heating to cooling upon isenthalpic throttling, i.e., $(\frac{\partial T^{*}}{\partial p^{*}})_{h^{*}} = 0$ also holds on the Charles curve. The Charles curve originates on the vapor pressure curve. The Charles curve ends at the Charles temperature $T_\text {Charles}^{*}$ in the zero-pressure limit $p^{*} \rightarrow 0$, which corresponds to the condition for the second virial coefficient $\text {d} B^{*} / \text {d}T^{*} = B^{*}/T^{*}$ (the secant of the second virial coefficient at $B^{*}(T_\text {Charles}^{*})$ is a line through the origin) [39]. This relation can be straightforwardly derived by applying $\big ( \frac{\partial Z}{\partial T^{*}} \big )_{p^{*}} = 0$ to the pressure-based virial equation in the low-density limit where the third and higher virial coefficients are negligible [74].

Furthermore, it has been shown that the terminal slope of the characteristic curves at high temperatures are related to both the second and third virial coefficient [74], which follows from the nature of the virial expansion.

Details and alternative thermodynamic definitions for the characteristic curves can be found in Refs. [39, 70, 73, 75, 77].

The second and third virial coefficient $B^{*}$ and $C^{*}$, respectively, of a fluid can be directly computed from the pairwise additive intermolecular potential, e.g., the LJ potential $u_\text {LJ}$ [78, 79]. Using the Mayer function

8 $$\begin{aligned} f_{ij}^{*} = \exp \big ( - u_\text {LJ}^{*}(r_{ij}^{*})/T^{*} \big ) - 1 \, , \end{aligned}$$

where $r_{ij}^{*}$ indicates the distance between two interacting particles, the second and third virial coefficient can be written as [79]

9 $$\begin{aligned} B^{*}= & {} - \frac{1}{2V^{*}} \iint f_{12}^{*} \, \text {d}{\mathbf {r}}_1^{*} \text {d}{\mathbf {r}}_2^{*} \, , \end{aligned}$$

10 $$\begin{aligned} C^{*}= & {} - \frac{1}{3V^{*}} \iiint f_{12}^{*} \, f_{23}^{*} \, f_{13}^{*} \, \text {d}{\mathbf {r}}_1^{*} \text {d}{\mathbf {r}}_2^{*} \text {d}{\mathbf {r}}_3^{*} \, , \end{aligned}$$

where $\text {d}{\mathbf {r}}^{*}$ indicates a finite volume element in which the particle is located. Equation (9) can be integrated in a trivial way, cf. Ref. [79]. The integrals in Eq. (10) were solved in this work using the method proposed by Hutem and Boonchui [80]. Equations (9) and (10) were implemented and numerically integrated to obtain exact results (within the significant digits used for the computer precision in the calculations) for $B^{*}$ and $C^{*}$ as well as their characteristic points using the LJ potential. ’Exact’ means here that no statistical uncertainty applies to the data (in contrast to molecular simulation results).

Furthermore, the second and third viral coefficient were computed from the considered LJ EOS, cf. Table 1. Equations (11) and (12) give the thermodynamic definitions for the calculation of the second and third virial coefficient $B^{*}$ and $C^{*}$ from the Helmholtz energy:

11 $$\begin{aligned} B^{*}= & {} \lim \limits _{\rho ^{*} \rightarrow 0 } \bigg (\frac{\partial (p^{*}/\rho ^{*} T^{*}) }{\partial \rho ^{*}}\bigg )_{T^{*}} = \rho ^{*-1} \lim \limits _{\rho ^{*} \rightarrow 0 } ( {\tilde{a}}_\text {01}^{{*}\text {conf}}/ \rho ^{*} ) \, , \end{aligned}$$

12 $$\begin{aligned} C^{*}= & {} 1/2 \cdot \lim \limits _{\rho ^{*} \rightarrow 0 } \bigg (\frac{\partial ^2 (p^{*}/\rho^{*} T^{*}) }{\partial \rho ^{*2}}\bigg )_{T^{*}} = \rho ^{{*} -2} \lim \limits _{\rho ^{*} \rightarrow 0 } ( {\tilde{a}}_\text {02}^{{*}\text {conf}}/ \rho ^{*2} ) \, . \end{aligned}$$

## Results

### Virial Coefficients

**Fig. 1:**
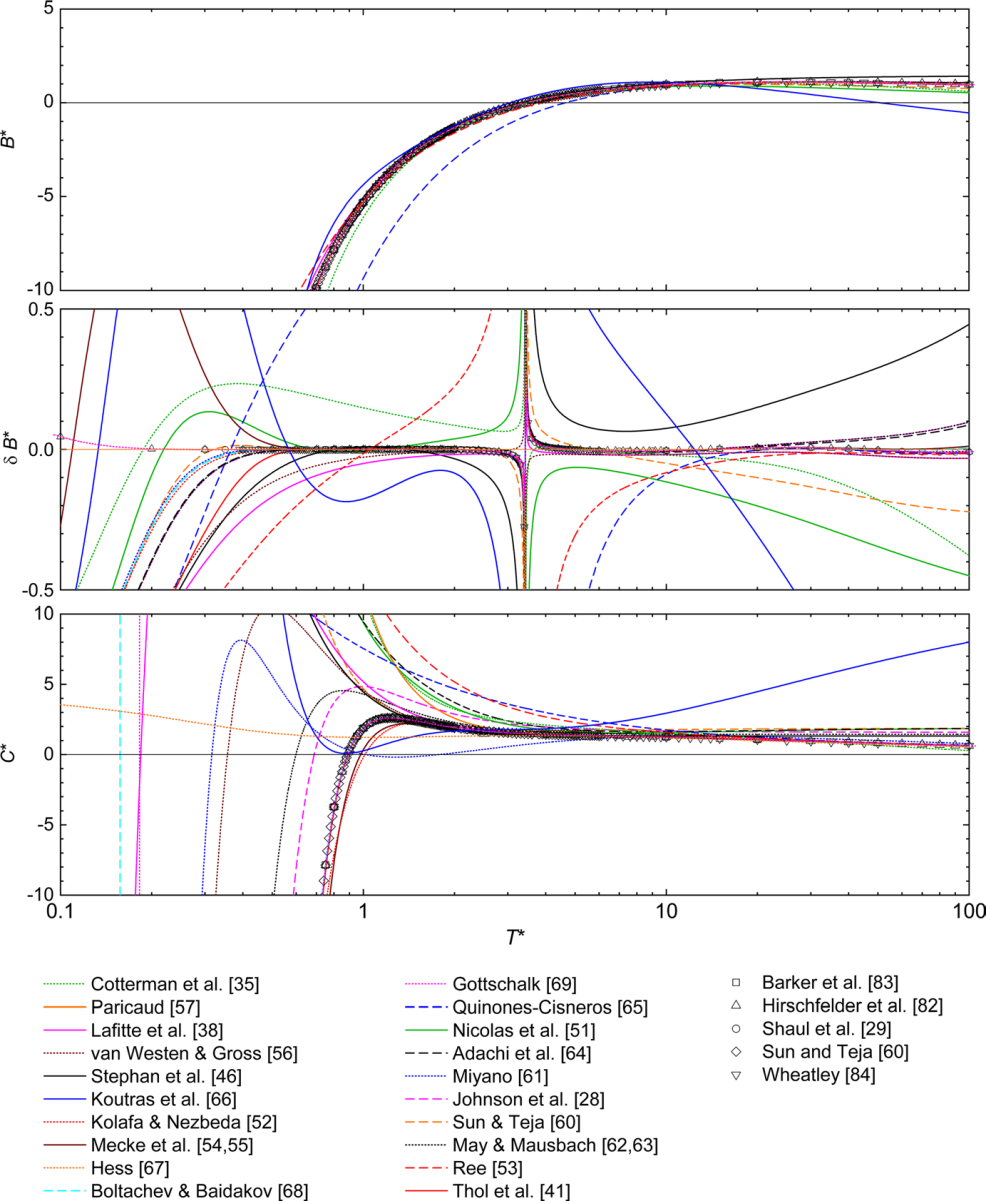
Second (top and middle) and third (bottom) virial coefficient as a function of the temperature. The top and bottom plot show the virial coefficients themselves; the middle plot shows the relative deviation $\delta B^{*} = (B_\text {EOS}^{*}-B_\text {ref}^{*})/B_\text {ref}^{*}$ of the second virial coefficient from the LJ EOS of Paricaud [57], i.e., the baseline ’ref’ corresponds to Ref. [57]. For all three plots: lines are LJ EOS and exact values from Refs. [29, 60, 82–84] are symbols

The second and third virial coefficient computed from the 20 considered LJ EOS are compared in Fig. 1 with exact data obtained from statistical mechanics [81] published in the literature [7, 29, 60, 82–84]. Numbers from our implementation perfectly agree with that literature data. The literature values are plotted for reproducibility reasons. The numeric values for the second and third virial coefficient computed from the 20 considered LJ EOS are reported in the electronic Supplementary Material.

Qualitatively, the second virial coefficient $B^{*}$ of the LJ fluid is captured well by all considered LJ EOS, cf. Fig. 1—top, except that of Koutras et al. [66]. All other LJ EOS have a single zero crossing at the Boyle temperature $T_\text {Boyle}^{*}$ (defined as $B^{*}(T_\text {Boyle}^{*})=0$). The exact value of the Boyle temperature obtained from numerical integration is $T_\text {Boyle}^{*} = 3.417927982$. However, significant deviations from the exact second virial coefficient data are found for most LJ EOS at low temperatures (below the triple point temperature, which is approximately $T_\text {tr}^{*} = 0.68\pm 0.02$ [85–92]) and at high temperatures ($T^{*}>10$), cf. Fig. 1—middle. Excluding the direct vicinity of the Boyle temperature, all LJ EOS except that of Paricaud [57], Gottschalk [69], and Hess [67] show relative deviations from the exact values for the second virial coefficient of at least 20% in some temperature range. The LJ EOS of Refs. [57, 67, 69] comprise the statistical mechanical formulation for the second virial coefficient in their mathematical formulation, which consequently leads to an excellent agreement for $B^{*}$. However, minor deviations for $B^{*}$ are observed for the results obtained from our implementation of the LJ EOS of Paricaud [57] at low temperatures.

The LJ EOS of Quiñones-Cisneros et al. [65], Nicolas et al. [51], Cotterman et al. [35], Sun and Teja [60], Koutras et al. [66], and Stephan et al. [46] deviate by more than 20% from the exact values in the range $T^{*}<T_\text {tr}^{*}$ and $6<T^{*}$. The LJ EOS of van Westen and Gross [56], Lafitte et al. [38], Thol et al. [41], Adachi et al. [64], May and Mausbach [62, 63], Johnson et al. [28], Kolafa and Nezbeda [52], Boltachev and Baidakov [68], Ree [53], and Miyano [61] deviate by more than 20% from the exact values at $T^{*}<T_\text {tr}^{*}$, too, but perform better at high temperatures. Excluding the vicinity of the Boyle temperature and extreme temperature conditions at $T^{*}<T_\text {tr}^{*}$ and $6<T^{*}$, the LJ EOS of Mecke et al. [54, 55], Johnson et al. [28], Boltachev and Baidakov [68], Adachi et al. [64], Miyano [61], Thol et al. [41], and Kolafa and Nezbeda [52] describe the exact second virial coefficient data within $\delta B^{*} = \pm 2\%$—the LJ EOS of Refs. [41, 52, 54, 55, 64, 68] even better than 0.5% in a wide temperature range.

The agreement of the LJ EOS and exact values for the third virial coefficient $C^{*}$ is overall significantly less good than for the second virial coefficient. Only the LJ EOS of Johnson et al. [28], Kolafa and Nezbeda [52], Lafitte et al. [38], Mecke et al. [54, 55], May and Mausbach [62, 63], Thol et al. [41], and van Westen and Gross [56] qualitatively describe the trend of the third virial coefficient accurately. The LJ EOS of Kolafa and Nezbeda [52], Mecke et al. [54, 55], and Thol et al. [41] describe the third virial coefficient qualitatively well up to $T^{*}=100$. The absolute average deviations from these three LJ EOS and the exact values for $C^{*}$ from the literature (Refs. [7, 29, 60, 82–84, 93]) are $\text {AAD}^\text {Me} = 0.47$, $\text {AAD}^\text {Ko} = 0.53$, and $\text {AAD}^\text {Th} = 0.08$, i.e., that of Thol et al. [41] is the most accurate regarding the description of $C^{*}$. Also the results from the LJ EOS of Boltachev and Baidakov [68] and Gottschalk [69] are in very good agreement with the exact data for the third virial coefficient at moderate and high temperatures (cf. Fig. 1—bottom), too, but both yield a wrong limit at low temperatures.

Other considered LJ EOS either exhibit no maximum or two maxima or a wrong limit at low temperatures. Even though the LJ EOS of Refs. [57, 67, 69] were found to be the most precise LJ EOS to describe the second virial coefficient, these LJ EOS produce a qualitatively wrong trend for the third virial coefficient. As it is possible to discriminate between the LJ EOS based on their ability to reproduce $B^*(T^*)$ and particularly $C^*(T^*)$, no attempt was made to discuss their predictions of $D^*(T^*)$ and higher virial coefficients.

Castro-Marcano et al. [94] showed that theoretically based EOS, such as soft-SAFT [36, 37], SAFT-VR [38, 49], and PC-SAFT [50, 95] do not adequately describe third virial coefficients of real substances in a sense that they do not exhibit a maximum at moderate temperatures and wrong limits at low temperatures. Our results indicate that for the PC-SAFT equation, this deficiency is already inherent in the corresponding monomer term (the LJ EOS of Ref. [46]), whereas the soft-SAFT and SAFT-VR Mie equation show a physically correct behavior for monomers.

### Characteristic Curves

Multiple computer experiment data sets for the characteristic curves of the LJ fluid are available in the literature [75, 96–100]. The Charles curve (a.k.a. Joule–Thomson inversion curve) of the LJ fluid has been investigated several times in the literature by molecular simulations [75, 96–100]. The Amagat, Boyle, and Zeno curve of the LJ fluid have only been investigated by computer experiment by Deiters and Neumaier [75]. The numeric values of these computer experiment data were summarized in Ref. [7] and are taken here as reference.

**Fig. 2:**
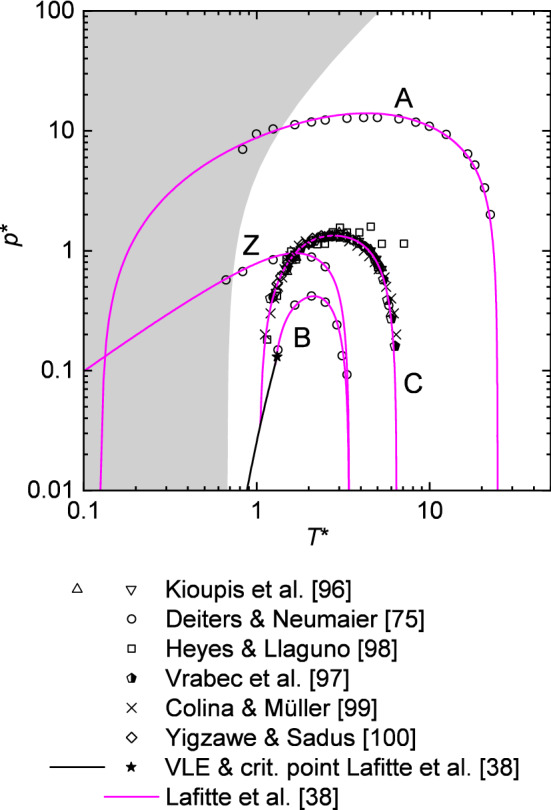
Brown’s characteristic curves: lines are the LJ EOS of Lafitte et al. [38]; symbols are molecular simulations results from the literature [75, 96–100]. The black solid line and star indicate the VLE and critical point. The gray-shaded region indicates the solid phase of the LJ potential as reported by Agrawal and Kofke [89]

The computer experiment data available for the characteristic curves are compared in Fig. 2 with the results obtained from the LJ EOS of Lafitte et al. [38], which gives the best description of the characteristic curve reference data (discussed in detail below). The Charles curve computer experiment data points of Refs. [75, 96, 97, 99, 100] are in good mutual agreement. For the Charles curve, the computer experiment data reported by Heyes and Llaguno [98] is found to deviate significantly from the data of Refs. [75, 96, 97, 99, 100]. To avoid visual clutter, only the data of Deiters and Neumaier [75] is used in the following for the evaluation of the LJ EOS. Figure 2 also shows the solid–fluid transition reported by Agrawal and Kofke [89].

It turns out that some of the simulation data of Deiters and Neumaier [75] for the Amagat and Charles curves probably lie beyond the freezing line. It is well known, however, that small simulation ensembles in cubic boxes with periodic boundary conditions tend to supercool. Deiters and Neumaier [75] used a moderate ensemble size of $N = 1000$ particles, they always started their simulations from random configurations, and they monitored the simulations runs for signs of crystallization. One can therefore conclude that the reported simulation states beyond the freezing line represent supercool fluid states.

Figure 2 shows that the four characteristic curves computed from the LJ EOS of Lafitte et al. [38] satisfies all requirements postulated by Brown [70], i.e., starting points on the vapor pressure curve, limits at $p^{*} \rightarrow 0$, and intersection points, except the termination point of the Zeno and Amagat curve. Brown deduced from rational thermodynamic arguments that the Zeno and Amagat curve converge into each other in the zero-temperature and zero-pressure limit with infinite slope. However, it is interesting to note that the LJ EOS of Lafitte et al. [38] yields a crossing of the Zeno and Amagat curves at approximately the critical pressure. Furthermore, the Zeno curve of the LJ EOS of Lafitte et al. [38] does not exhibit an infinite slope in the zero-pressure limit as postulated by Brown [70].

Brown’s [70] assumption for an infinite slope of the Zeno curve in the zero-pressure limit at low $T^{*}$ is probably incorrect. The compressibility factor on the Zeno curve is by definition $Z=1$, which yields $\ln p^{*} = \ln T^{*} + \ln \rho ^{*}$. The last term converges approximately to a constant value at low $p^{*}$ for $T^{*}\rightarrow 0$. Hence, the Zeno curve has a constant slope of unity at $T^{*}\rightarrow 0$ in a double-logarithmic $p^{*}-T^{*}$ diagram—as predicted by the LJ EOS of Lafitte et al. [38] and others (see below). Nonetheless, for the LJ model that region lies in the solid region.

**Fig. 3:**
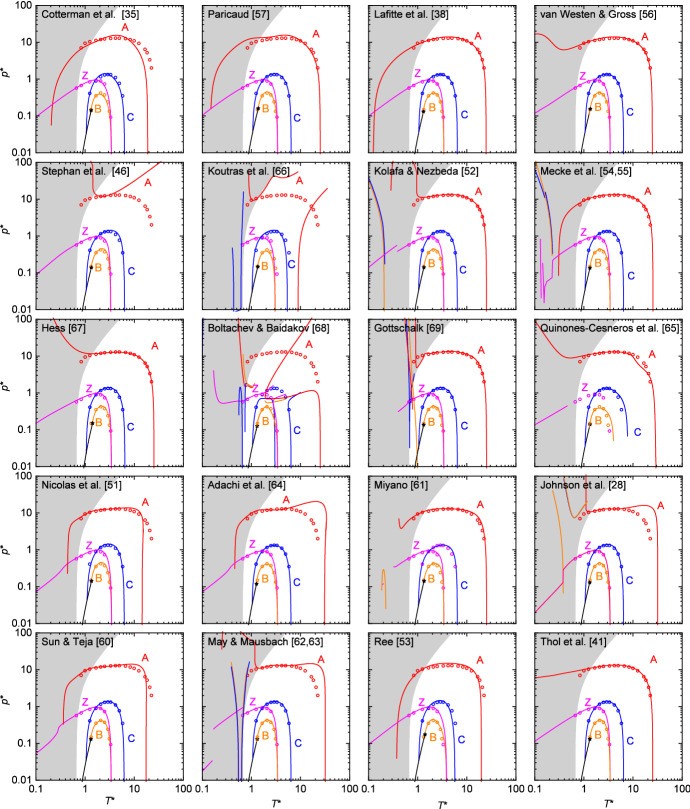
Comparison of Brown’s characteristic curves obtained from different LJ EOS (colored lines) with the molecular simulations results (symbols) of Deiters and Neumaier [75]. The black solid line and star indicate the VLE and critical point obtained from the respective LJ EOS. The gray-shaded region indicates the solid phase of the LJ potential as reported by Agrawal and Kofke [89]

The characteristic curve computer experiment data of Deiters and Neumaier [75] are compared in Fig. 3 individually with the results obtained from the 20 investigated LJ EOS. The LJ EOS are ordered roughly according their types, i.e., starting from the physically motivated LJ EOS, to the semi-theoretical and fully empirical LJ EOS.

None except the LJ EOS of Lafitte et al. [38] satisfies all requirements for the characteristic curves and is in good quantitative agreement with available computer experiment data. The characteristic curves obtained from the LJ EOS of Ree [53] are in accordance with Brown’s postulates, but show significant deviations from the computer experiment data. Most LJ EOS yield reasonable Zeno, Boyle, and Charles curves, but fail for the Amagat curve. In the case of inaccurate Zeno, Boyle, and Charles curves, they are mostly distorted at low temperatures. Several LJ EOS [41, 52, 56, 61, 67, 69] produce reasonable Amagat curves over a wide temperature range, but yield distortions in the vicinity of the solid–fluid equilibrium. There are also some LJ EOS that produce Amagat curves exhibiting minor oscillations at high pressures [28, 35, 51, 57, 60, 62–65], i.e., a wrong curvature.

A constant slope of unity for the Zeno curve at $T^{*}\rightarrow 0$ in the double-logarithmic $p^{*}-T^{*}$ diagram is also obtained from the LJ EOS of Refs. [35, 41, 46, 53, 56, 57, 67], cf. Fig. 3. This corroborates our argument concerning the original postulates of Brown [70].

The four characteristic curves studied here evidently represent challenges of different severity, i.e., the Charles curve is predicted qualitatively correct by most LJ EOS, while the Amagat curve is predicted qualitatively correct and in good agreement with reference data in the entire temperature range by merely one LJ EOS. There is a tendency among the four characteristic curves to be predicted qualitatively correct (Charles $\rightarrow$ Boyle $\rightarrow$ Zeno $\rightarrow$ Amagat).

Boshkova and Deiters [77] reported that many theory-based EOS fail to yield accurate Amagat curves due to simplifications in the modeling of the repulsive interactions. However, we find that the theory-based LJ EOS of Lafitte et al. [38], Cotterman et al. [35], and Paricaud [57] yield reasonable Amagat curves in a wide temperature range—the LJ EOS of Lafitte et al. [38] is even quantitatively in good agreement with computer experiment results.

The LJ EOS of Stephan et al. [46] based on the PC-SAFT monomer model yields qualitatively accurate Zeno, Boyle, and Charles curves, but a completely distorted Amagat curve. This type of behavior was also reported by Boshkova and Deiters [77] for the characteristic curves of the original PC-SAFT parametrization. They showed that this is a result of the simplified temperature-dependent diameter of the PC-SAFT approach which gives a poor description of the soft repulsion of the Lennard-Jones potential [77]. This is supported by the results from a recent work of our group [46], which showed that the LJ EOS of Stephan et al. (re-parametrized PC-SAFT monomer term) yields large deviations at high temperatures and densities for most homogeneous state point properties, where the softness of the repulsive interactions becomes more important.

The LJ EOS of the MBWR type (Refs. [28, 51, 60–64]) have in common that they yield Zeno curves with a kink at low temperatures. Some of them also exhibit a distorted Boyle curve (LJ EOS of Refs. [28, 61–63]). All LJ EOS of the MBWR type yield distorted Amagat curves, but most yield accurate Charles curves. For some MBWR type LJ EOS [51, 60, 64], the Amagat curve has a positive curvature at high $T^{*}$.

The LJ EOS of Cotterman et al. [35], Paricaud [57], Thol et al. [41], Hess [67], and van Westen and Gross [56] produce qualitatively accurate Zeno, Boyle, and Charles curves, but distorted Amagat curves—especially at low temperatures. The LJ EOS of Stephan et al. [46] (re-parametrized PC-SAFT monomer), Koutras et al. [66], and Boltachev and Baidakov [68] yield erratic results for the Amagat curve. The LJ EOS of Cotterman et al. [35] and Paricaud [57] have a faint bump at high temperatures, i.e., wrong curvature. The LJ EOS of Boltachev and Baidakov [68] and Quiñones-Cisneros et al. [65] show a distorted shape for all four characteristic curves. For the LJ EOS of Quiñones-Cisneros et al. [65], the Zeno curve at moderate temperatures lies below $p^{*}=0.01$, i.e., out of the range of the depicted plot. The characteristic curves obtained from the LJ EOS of Gottschalk [69], Kolafa and Nezbeda [52], and Mecke et al. [54, 55] are in good agreement with the reference data in a wide temperature and pressure range of the fluid region, but all four curves yield unrealistic solutions at low temperatures (for the Amagat curve of Ref. [54, 55] at $T^{*}<0.1$). Overall, for most LJ EOS the identified deficiencies are found in the vicinity and beyond the solid–fluid equilibrium, i.e., the Zeno and Amagat curves, whereas a reasonable performance is often found at high temperatures. It should be noted that state points beyond the solid–fluid equilibrium can also be relevant for the modeling of fluid mixtures.

The characteristic curves obtained from the LJ EOS of Mecke et al. [54, 55] are in excellent agreement with the available computer experiment data, but show unphysical features in the low-temperature limit. Deiters and Neumaier [75] reported that the LJ EOS of Mecke et al. [54, 55] gives a realistic description of all characteristic curves, which is found differently in the present work. An additional (physically unrealistic [70, 77]) branch is found for all four characteristic curves. Likewise, an unrealistic behavior is found for the Charles, Boyle, Zeno, and Amagat curve of the LJ EOS of Kolafa and Nezbeda [52] at low temperature.

The Amagat curve of the LJ EOS of Thol et al. [41] is distorted at lower temperatures—as also pointed out by Thol et al. [41] and Deiters and Neumaier [75]. However, we find a significantly different Amagat curve as reported by Thol et al. [41] for their LJ EOS. The Amagat curve computed from our implementation is in good agreement with the computer experiment results reported by Deiters and Neumaier [75] for most temperatures. We suspect a misprint in the publication of Thol et al. [41].

### Characteristic State Points

The thermodynamic behavior of a pure substance contains multiple uniquely defined state points, of which the critical point is the most prominent one. Such state points can be favorably used to characterize the quality of EOS, since they comprise a condensed description of the thermodynamic behavior in a single state point. The critical point obtained from different LJ EOS in comparison to computer experiment data has been discussed in detail elsewhere [7, 46]. Here, characteristic state points related to the virial coefficients and Brown’s characteristic curves are discussed. In particular, exact values for a given interaction potential can be obtained for some characteristic points from statistical mechanics.

**Fig. 4:**
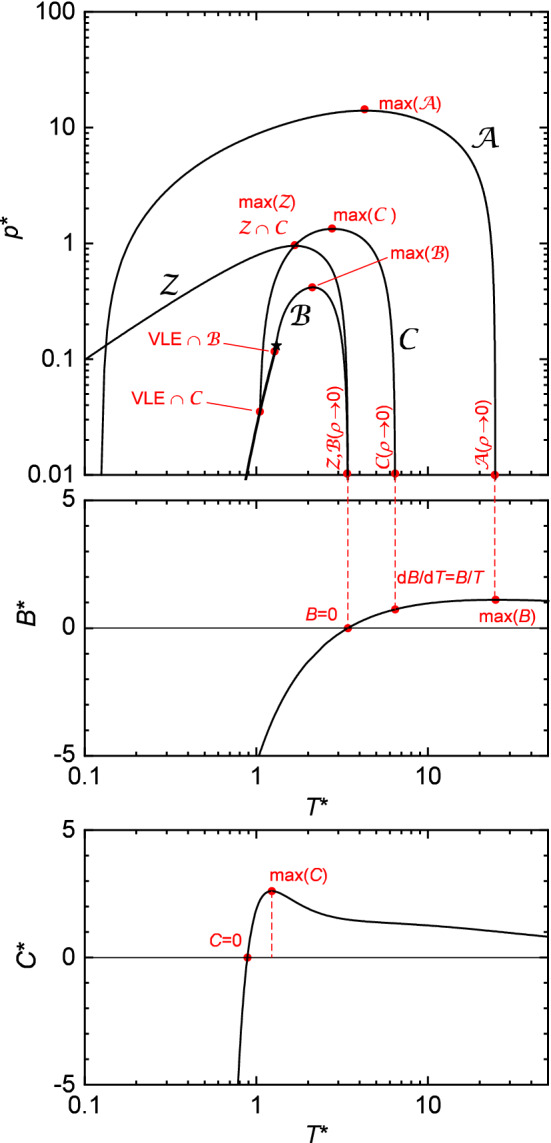
Scheme of the characteristic curves (top) and second and third virial coefficient (middle and bottom, respectively) for the illustration of the considered characteristic state points (red symbols), cf. Table 3

The characteristic state points considered in the present work are schematically illustrated in Fig. 4; they are defined as:

- the state points of the four characteristic curves in the zero-density limit, labeled as ${{\mathcal {Z}}(\rho ^{*} \rightarrow 0 )}$, ${{\mathcal {A}}(\rho ^{*} \rightarrow 0 )}$, ${{\mathcal {B}}(\rho ^{*} \rightarrow 0 )}$, and ${{\mathcal {C}}(\rho ^{*} \rightarrow 0 )}$ (which can also be computed from the virial coefficients—see above),
- the zero crossing of the third virial coefficient $C^{*}(T^{*})=0$, and the maximum of the third virial coefficient ${\text {max}({C^{*}(T^{*})})}$,
- the intersection of the Boyle and Charles curve with the vapor pressure curve labeled as ${\text {VLE}\, \cap \, {\mathcal {B}}}$ and ${\text {VLE}\, \cap \, {\mathcal {C}}}$,
- the intersection point of the Zeno and Charles curve labeled as ${{\mathcal {Z}}\, \cap \, {\mathcal {C}}}$,
- the maxima of the four characteristic curves in the $p^{*}-T^{*}$ plane labeled as ${ {\max}({\mathcal {A}})}$, ${{\max}({\mathcal {B}})}$, ${{\max}({\mathcal {C}})}$, and ${{\max}({\mathcal {Z}})}$.

Exact values from numerical integration of the virial coefficients can be obtained for the temperature at ${{\mathcal {Z}}(\rho ^{*} \rightarrow 0 )}$, ${{\mathcal {A}}(\rho ^{*} \rightarrow 0 )}$, ${{\mathcal {B}}(\rho ^{*} \rightarrow 0 )}$ from the second virial coefficient (cf. Fig. 4—top and middle), the zero crossing of the third virial coefficient $C^{*}(T^{*})=0$, and the maximum of the third virial coefficient ${\text {max}({C^{*}(T^{*})})}$ as indicated in Table 3 (cf. Fig. 4—bottom). These results are termed as ’results from the virial route’ in the following. Those reported values in Table 3 were obtained in this work and were—where available—compared and found in excellent agreement with results from the literature [101].

**Table 3:** Temperatures of characteristic state points calculated from different LJ EOS

	$T^{*}_{{\max}({\mathcal {Z}})}$	$T^{*}_{{\max}({\mathcal {B}})}$	$T^{*}_{{\max}({\mathcal {C}})}$	$T^{*}_{{\max}({\mathcal {A}})}$	$T^{*}_{\text {VLE} \cap {\mathcal {B}}}$	$T^{*}_{\text {VLE} \cap {\mathcal {C}}}$	$T^{*}_{{\mathcal {Z}} \cap {\mathcal {C}}}$	$T^{*}_{{\mathcal {Z}}(\rho ^{*} \rightarrow 0 )}$	$T^{*}_{{\mathcal {B}}(\rho ^{*} \rightarrow 0 )}$	$T^{*}_{{\mathcal {C}}(\rho ^{*} \rightarrow 0 )}$	$T^{*}_{{\mathcal {A}}(\rho ^{*} \rightarrow 0 )}$	$T^{*}_{C^{*} = 0}$	$T^{*}_{{\max}(C^{*})}$
Cotterman et al. [35]	1.6310	1.9335	2.5495	4.3358	1.3005	1.0572	1.6310	3.4321	3.4321	6.3469	18.7773	–	–
Paricaud [57]	1.7642	2.0918	2.9776	4.7771	1.3469	1.0651	1.7642	3.4248	3.4248	6.4571	25.1718	–	–
Lafitte et al. [38]	1.6711	2.1382	2.7890	4.2976	1.2838	1.0525	1.6711	3.4220	3.4220	6.4379	24.6615	0.1838	0.2704
van Westen and Gross [56]	1.7026	2.0406	2.8827	4.0655	1.2029	1.0579	1.7026	3.4280	3.4280	6.4444	24.6640	0.3559	0.4994
Stephan et al. [46]	1.7884	2.2171	3.3279	–	1.3008	1.0505	1.7884	3.3172	3.3172	6.3423	–	–	–
Koutras et al. [66]	1.5278	1.9851	2.8420	–	1.3187	1.0343	1.5278	3.0549	3.0549	5.3955	8.8877	–	–
Kolafa and Nezbeda [52]	1.7437	2.1305	2.9641	4.5240	1.3037	1.0448	1.7437	3.4176	3.4176	6.4303	25.1215	1.0110	1.5316
Mecke et al. [54, 55]	1.7326	2.1531	2.8817	4.4880	1.3058	1.0535	1.7326	3.4181	3.4181	6.4315	25.4520	0.9791	1.4068
Hess [67]	1.7466	2.1710	2.9804	4.6380	1.3789	1.0526	1.7466	3.4179	3.4179	6.4308	25.1526	–	–
Boltachev and Baidakov [68]	–	2.0292	–	–	1.1894	1.0538	2.8050	3.4183	3.4183	6.4339	25.2259	–	–
Gottschalk [69]	1.7392	2.1185	2.9255	4.6550	1.2959	1.0497	1.7393	3.4179	3.4179	6.4308	25.1526	–	–
Quiñones-Cisneros et al. [65]	1.7456	2.0612	2.8011	4.1177	1.3029	1.0570	1.7456	–	–	–	38.0169	–	–
Nicolas et al. [51]	1.8031	2.1744	3.0167	7.3349	1.3281	1.0998	1.8031	3.4748	3.4748	6.2763	14.4852	–	–
Adachi et al. [64]	1.6818	2.0867	2.8610	20.3570	1.2570	1.0373	1.6818	3.4148	3.4148	6.4090	30.9385	–	–
Miyano [61]	1.7547	2.2564	3.2542	3.6436	1.3165	1.0349	1.7547	3.4151	3.4151	6.4166	25.1903	0.3159	0.3938
Johnson et al. [28]	1.7321	2.1165	3.0488	16.7742	1.2838	1.0567	1.7321	3.4150	3.4150	6.4147	31.8491	0.6878	0.9906
Sun and Teja [60]	1.6831	2.1198	2.8851	8.6988	1.2858	1.0506	1.6831	3.4004	3.4004	6.2288	17.4236	–	–
May and Mausbach [62, 63]	1.7267	2.1387	3.0118	15.9232	1.2859	1.0617	1.7267	3.4150	3.4150	6.4147	31.8491	0.5986	0.8588
Ree [53]	1.7771	2.0370	2.9266	3.8508	1.3725	1.0894	1.7771	3.8746	3.8746	7.3462	28.5118	–	–
Thol et al. [41]	1.7424	2.1760	2.9211	4.4841	1.2943	1.0512	1.7424	3.4168	3.4168	6.4253	25.1728	0.8884	1.2139

The columns are from left to right: the maxima of the four characteristic curves, the intersection point of the Boyle and Charles curve with the VLE phase boundary, the intersection point of the Zeno and Charles curve, the zero-density limit of the four characteristic curves, the zero crossing of the third virial coefficient, and the maximum of the third virial coefficient. The full specifications of the state points are reported in the electronic Supplementary Material. Exact values obtained from the virial coefficient route by numerical integration; Eqs. (9) and (10), cf. Fig. 4—middle and bottom: $T^{*}_{C^{*} = 0} = 0.886917868$, $T^{*}_{\text {max}(C^{*})} = 1.229875759$, $T^{*}_{{\mathcal {B}}(\rho ^{*} \rightarrow 0 )} = 3.417927982$, $T^{*}_{{\mathcal {C}}(\rho ^{*} \rightarrow 0 )} = 6.430798418$, $T^{*}_{{\mathcal {A}}(\rho ^{*} \rightarrow 0 )} = 25.15242837$

Furthermore, the above-defined characteristic state points were computed for each of the considered LJ EOS. For the maxima and intersection points of the characteristic curves, an iterative solver was used to find the state point satisfying the respective conditions. For the intersection points of the characteristic curves with the phase envelope, both the VLE and the characteristic curves were iteratively computed by means of a given LJ EOS to find the intersection point. The zero-density limit state points of the characteristic curves were computed directly from the definition of the respective curve at $\rho ^{*} \rightarrow 0$. To validate the consistency of the LJ EOS implementations used in the present work, these state points were also computed by the LJ EOS from the corresponding definition from the second virial coefficient (see above) for comparison. The results obtained from the two thermodynamic definitions were found in all cases to be in perfect agreement.

Table 3 lists the temperatures of the characteristic state points. The temperature, pressure, and density of each state point are reported in the electronic Supplementary Material. The numeric values therein are reported with more decimal places than in Table 3. Blanks in Table 3 indicate cases where the shape of a characteristic curve or the third virial coefficient is distorted in a way that a maximum or crossing point could not be evaluated in a meaningful way.

The temperatures $T^{*}_{{\mathcal {Z}}(\rho ^{*} \rightarrow 0 )}$, $T^{*}_{{\mathcal {B}}(\rho ^{*} \rightarrow 0 )}$, $T^{*}_{{\mathcal {C}}(\rho ^{*} \rightarrow 0 )}$, and $T^{*}_{{\mathcal {A}}(\rho ^{*} \rightarrow 0 )}$ (zero-density limit state points of the characteristic curves) obtained from the LJ EOS can be compared with exact results computed by numerical integration via the virial coefficient route, cf. Fig. 4—middle and Table 3. Excellent agreement is found for many LJ EOS except the LJ EOS of Refs. [46, 51, 53, 56, 60, 65, 66] which yield significantly deviating $T^{*}_{{\mathcal {Z}}(\rho ^{*} \rightarrow 0 )}$ and $T^{*}_{{\mathcal {B}}(\rho ^{*} \rightarrow 0 )}$; the LJ EOS of Refs. [28, 35, 46, 51, 53, 60, 62–66] yield significantly deviating $T^{*}_{{\mathcal {A}}(\rho ^{*} \rightarrow 0 )}$ (or even no solution).

The zero-density limits $T^{*}_{{\mathcal {Z}}(\rho ^{*} \rightarrow 0 )}$, $T^{*}_{{\mathcal {B}}(\rho ^{*} \rightarrow 0 )}$, $T^{*}_{{\mathcal {C}}(\rho^{*} \rightarrow 0 )}$, and $T^{*}_{{\mathcal {A}}(\rho ^{*} \rightarrow 0 )}$ obtained from the LJ EOS of Gottschalk [69] and Hess [67] agree with the exact values within the computer precision employed for the calculations since those are integrated in the respective equation. For the LJ EOS of Paricaud [57] small deviations for the $T^{*}_{{\mathcal {Z}}(\rho ^{*} \rightarrow 0 )}$, $T^{*}_{{\mathcal {B}}(\rho ^{*} \rightarrow 0 )}$, $T^{*}_{{\mathcal {C}}(\rho ^{*} \rightarrow 0 )}$, and $T^{*}_{{\mathcal {A}}(\rho ^{*} \rightarrow 0 )}$ in comparison to the exact data are found, which is in line with the small deviations observed for the second virial coefficient itself. Also the LJ EOS of Kolafa and Nezbeda [52] has a second virial coefficient term and therefore gives an excellent description of the zero-density limits of the characteristic curves. Also the empirical LJ EOS of Thol et al. [41] yields accurate results for $T^{*}_{{\mathcal {Z}}(\rho ^{*} \rightarrow 0 )}$, $T^{*}_{{\mathcal {B}}(\rho ^{*} \rightarrow 0 )}$, $T^{*}_{{\mathcal {C}}(\rho ^{*} \rightarrow 0 )}$, and $T^{*}_{{\mathcal {A}}(\rho ^{*} \rightarrow 0 )}$ (deviations below 2%).

Exact values were also obtained in the present work for the temperature of the zero crossing and maximum of the third virial coefficient $T^{*}_{C^{*}=0}$ and $T^{*}_{\text {max}({C^{*}})}$ from numerical integration, cf. Table 3. Only results from the LJ EOS of Refs. [41, 52, 54, 55] are found to be in good agreement with exact values for $T^{*}_{C^{*}=0}$ and $T^{*}_{\text {max}({C^{*}})}$; reasonable agreement is found for the LJ EOS of Refs. [28, 62, 63]. The best results for $T^{*}_{C^{*}=0}$ and $T^{*}_{\text {max}({C}^{*})}$ are obtained from the LJ EOS of Thol et al. [41].

The temperature of the Zeno curve maximum $T^{*}_{\max({\mathcal {Z}})}$ obtained from the 20 investigated LJ EOS are in good agreement. They lie in the range $T^{*}_{\max({\mathcal {Z}})} = 1.74 \pm 0.06$. The temperature of the Boyle curve maximum obtained from the 20 LJ EOS scatters slightly more in the range $T^{*}_{\max({\mathcal {B}})} = 2.12 \pm 0.09$. Only the LJ EOS of Koutras et al. [66] yields a significantly lower $T^{*}_{\max({\mathcal {Z}})}$ and $T^{*}_{\max({\mathcal {B}})}$ compared to all other investigated LJ EOS. The scattering is significantly larger for the temperature of the Charles curve maximum obtained from the different LJ EOS as $T^{*}_ {\max({\mathcal {C}})} = 2.92 \pm 0.5$. For the temperature of the Amagat curve maximum, 11 LJ EOS scatter around $T^{*}_{\max({\mathcal {A}})} = 4.5 \pm 1.5$. The LJ EOS of Refs. [28, 35, 60, 62–64, 68] show significantly shifted $T^{*}_{\max({\mathcal {A}})}$ which is due to the distorted Amagat curves produced by these LJ EOS.

The differences in the intersection points of the Charles and Boyle curves with the VLE are dominated by differences in the VLE obtained from the different LJ EOS—especially close to the critical point. The temperature of the Zeno and Charles curves intersection point (corresponding to ${\max({\mathcal {Z}})}$) obtained from the considered LJ EOS agrees within $T^{*}_{{\mathcal {Z}}\, \cap \, {\mathcal {C}}} = 1.74 \pm 0.06$, excluding the LJ EOS of Ref. [68].

Sadus [101] recently reported values for the Boyle temperature and the maximum of the second virial coefficient computed from the LJ EOS of Koutras et al. [66], which significantly deviate from the values obtained from our implementation for that LJ EOS. However, we compared $B^{*}(T^{*})$ obtained from our implementation with results originally reported by Koutras et al. [66] and found excellent agreement.

## Conclusions

The present work revisits Brown’s characteristic curves and virial coefficients of the Lennard-Jones fluid. They were computed from a large number of LJ EOS and from rigorous statistical mechanics (where accessible). For most LJ EOS, these properties have not been examined yet—especially the physically motivated ones.

The second virial coefficient is predicted qualitatively correctly by all but one LJ EOS. However, significant quantitative deviations are observed for most considered LJ EOS. For the third virial coefficient, only few LJ EOS produce qualitatively correct results.

Brown’s characteristic curves [70] predicted from the different LJ EOS were compared with computer experiment data of Deiters and Neumaier [75] and with exact values in the ideal gas limit. The physically motivated LJ EOS are found to give an overall better description of the characteristic curves—especially at low temperature and high pressure. Most LJ EOS produce distorted Amagat curves. Only the LJ EOS of Lafitte et al. [38] yields realistic results for all characteristic curves in the entire temperature range that are also in good quantitative agreement with available computer experiment data. The LJ EOS of Ree [53] yields realistic descriptions for the characteristic curves, but significant deviation to computer experiment data. Nevertheless, in several cases, a reasonable performance is found in a wide temperature range, e.g., the LJ EOS of Refs. [35, 41, 56, 57, 67, 69].

We showed that Brown’s assumption that the Amagat and Zeno curves should converge with an infinite slope in the zero-pressure limit at low temperatures (in the double-logarithmic pressure–temperature diagram) is probably inaccurate. The Zeno curve exhibits a limiting slope of unity. Hence, the required intersection point of the Amagat and Zeno curves is not found in the zero-pressure limit.

Brown’s characteristic curves are found to be sensitive properties, in a sense that they clearly reveal unphysical behavior of an EOS, which holds in particular for the Amagat and Zeno curve. The Boyle and Charles curve are found to be predicted accurately by most LJ EOS and are therefore less sensitive indicators. Nevertheless, the application of the characteristic curves to investigate ’the extrapolation behavior of EOS’ [73] should be carried out with caution. For example, the characteristic curves from the LJ EOS of Mecke et al. [54, 55] and Lafitte et al. [38] are in good agreement with corresponding computer experiment data in a wide temperature range, but both LJ EOS exhibit large deviations from pressure and internal energy reference data at extreme temperature and density (beyond the Amagat curve), cf. Ref. [46]. Vice versa, the LJ EOS of Kolafa and Nezbeda [52] and Thol et al. [41] exhibit distorted Amagat curves at low temperatures, but both LJ EOS give an overall accurate and fairly precise description of pressure and internal energy reference data also at extreme conditions [46]. Hence, for these LJ EOS the findings for the performance on the characteristic curves could not be transferred to conditions significantly above the pressure and temperature range of the characteristic curves. Instead, it is emphasized that the characteristic curves are a necessary requirement for an EOS to be accurate in the entire temperature and pressure range, but not a sufficient one.

## Electronic supplementary material

Below is the link to the electronic supplementary material.

Supplementary material 1 (XLSX 1043 kb)
